# Electrodeposition of V-VI Nanowires and Their Thermoelectric Properties

**DOI:** 10.3389/fchem.2019.00516

**Published:** 2019-08-06

**Authors:** Cristina V. Manzano, Marisol Martin-Gonzalez

**Affiliations:** Instituto de Micro y Nanotecnología, IMN-CNM, CSIC (CEI UAM+CSIC), Madrid, Spain

**Keywords:** electrodeposition, Bi_2_Te_3_, nanowires, thermoelectric properties, Seebeck coefficient, electrical conductivity, thermal conductivity, anodic aluminum oxide

## Abstract

Nanostructuration is an intensive field of research due to the appearance of interesting properties at the nanoscale. For instance, in thermoelectricity the most outstanding improvements obtained lately are related to phenomena that appear as a result of nano-engineering different materials. The thermoelectric effect is the direct conversion from temperature gradients into electricity and *vice versa*. When going to low dimensions, for example in the particular case of thermoelectric nanowires, the transport properties of phonons are modified with respect to those found in bulk leading to a higher thermoelectric figure of merit *z*. In more detail, this review tries to compile some of the landmarks in the electrodeposition of Bi_2_Te_3_-based nanowires. We will focus on the achievements using different templates, electrolytes and deposition modes. We will also summarize the measurements performed in those nanowires and the main conclusions that can be extracted from the published works. Finally, an update of nanowire-based thermoelectric generators is also included.

## Introduction

Thermoelectric materials have received significant attention in the last decades given that these materials are able to transform a temperature gradient into electricity, and thus they provide a sustainable source of electrical energy wherever a heat source can be found. The efficiency of these materials depends on the figure of merit (*z*), which is given by the following equation:

(1) $$\begin{array}{l} z\cdot T=\frac{{\sigma \cdot S}^{2\;}}{\kappa }\cdot T, \end{array}$$

where *T* is the absolute temperature, σ is the electrical conductivity, *S* is the Seebeck coefficient and κ is the thermal conductivity. The thermoelectric efficiency (η) is defined as:

(2) $$\begin{array}{l} \eta \left(\%\right)=100\left(\frac{T_{H-}T_{C}}{T_{H}}\right)\frac{\sqrt{1+z\cdot T}-1}{\sqrt{1+z\cdot T}+\left(\frac{T_{H}}{T_{C}}\right)}, \end{array}$$

where *T*_*H*_ is the temperature at the hot side and *T*_*C*_ is the temperature on the cold side. Nowadays, the main drawback that these materials present is a lower efficiency when compared with other ways of obtaining electrical energy. In order to improve the efficiency of these materials, its figure of merit has to be increased.

Nanostructuration is one of the approaches to increase the figure of merit in thermoelectric materials (Dresselhaus et al., 2007; Martín-González et al., 2013; Ali et al., 2017; Chen et al., 2018; Goktas et al., 2018; Swinkels and Zardo, 2018; Selvan et al., 2019). In this sense, nanowires are a great field of study to underline the physics behind the influence of the nanostructuration on the improvement of the figure of merit (Domínguez-Adame et al., 2019). One of the parameters that is more influenced by nanostructuration is the lattice thermal conductivity. This is due to the higher surface to volume ratio that these structures present (Domínguez-Adame et al., 2019). At the surface of the nanowires phonons are scattered, and this produces a reduction of the lattice thermal conductivity. Taking into account that the figure of merit is inversely proportional to the thermal conductivity, a reduction of this parameter produces an increase in the thermoelectric efficiency. Moreover, the smaller the nanowire diameter, the larger the phonon dispersion and, thus, the higher the thermoelectric figure of merit (Borca-Tasciuc et al., 2004).

**Figure 1:**
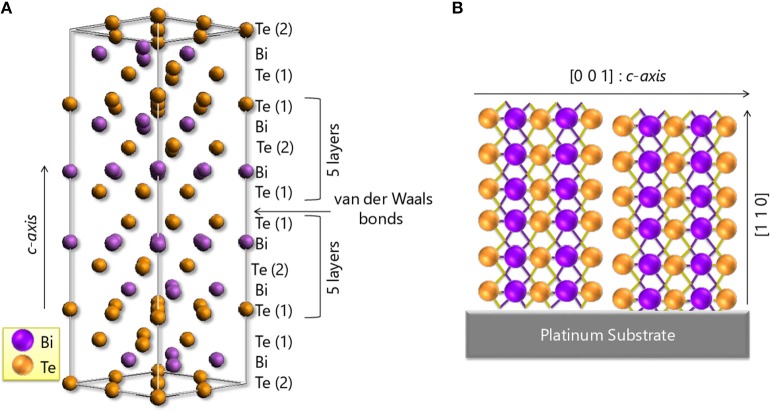
**(A)** Crystal structure of bismuth telluride. The structure shows the five atomic layers stacked along the c-axis (quintuplet) separated by van der Waals bonds. **(B)** The orientation of a Bi_2_Te_3_ electrodeposited film, which main diffraction peaks will be [110] related. As can be observed in this configuration the quintuplets are perpendicular to the substrate and so the van der Walls bonds. Reproduced with permission from Manzano et al. (2016a). Copyright 2016 Scientific Reports-Nature.

Some of the most studied thermoelectric materials are Bismuth Telluride-based. Its best efficiency is around room temperature. And, its ternary compounds are the ones most used in commercially available devices. Bi_2_Te_3_-based materials, apart from being the best thermoelectric for room temperature applications, belong also to a class of quantum materials called three-dimensional topological insulators (3D-TIs) (Hasan and Kane, 2010; Qi and Zhang, 2011). This quantum form of matter presents unique and topologically protected surface states (Zhang H. et al., 2009; Zhang T. et al., 2009; Chen et al., 2012; Muñoz Rojo et al., 2016); Apart from that, Bi_2_Te_3_ has a band gap of 0.15 eV (Greenaway and Harbeke, 1965); and a rhombohedral structure with space group $D_{3d}^{5}$ (R$\bar{3}$*m*), although it can also be described in hexagonal coordinates. The crystal structure has a layered disposition with five atomic layers with covalent bonding as the basic unit (cell), named as the quintuple layer (QL). The bonding between the QLs is much weaker than the inter-layer bonding since it is a van de Waals-type interaction. Due to this crystallographic structure (see Figure 1), this material presents high anisotropy in the electrical (Delves et al., 1961) and thermal conductivities (Goldsmid, 1961; Tritt and Subramanian, 2006), while the Seebeck coefficient is nearly isotropic (Rowe, 2012).

In more detail, the figure of merit of bulk single-crystalline bismuth telluride in the direction parallel to the *c-axis* of the structure was determined by Antonova et al. (Antonova and Looman, 2005). Its *zT*_//*c*_ is of 0.31, with Seebeck coefficient, electrical conductivity, and thermal conductivity values of −240 μV/K, 0.02 (μΩ·m)^−1^, and 1 W/m·K, respectively (Antonova and Looman, 2005). Conversely, when the crystal structure was oriented perpendicular to the *c-axis*, the values of the Seebeck coefficient, electrical conductivity, and thermal conductivity were −240 μV/K, 0.1 (μΩ·m)^−1^, and 2.2 W/m·K, respectively, which corresponds to a higher figure of merit of *zT*_⊥*c*_ = 0.78 (Antonova and Looman, 2005).

In the case of electrodeposited Bi_2_Te_3_ films, Martin-Gonzalez's group (Manzano et al., 2016a) reported the effect of anisotropy in highly oriented [110] Bi_2_Te_3_ electrodeposited films. The electrical conductivity perpendicular to the *c-axis* is nearly five (4.8) times higher than the electrical conductivity parallel the *c-axis*. The Seebeck coefficient perpendicular to the c-*axis* is within the experimental uncertainty of the Seebeck coefficient along the c-*axis*, indicating the electrodeposited film is isotropic for this property. A two-fold increase from the in-plane to out-of-plane thermal conductivity was observed. From the measured in-plane and out-of-plane values at 300 K, figure of merits of *zT*_//*c*_ = (5.6 ± 1.2)·10^−2^ and *zT*_⊥*c*_ = (10.4 ± 2.6)·10^−2^ are respectively rendered, which yields an increase by a factor of 1.8 between the in-plane and out-of-plane thermoelectric performances (Manzano et al., 2016a). Because of this anisotropy, it is very important to achieve stoichiometric nanowire arrays oriented along [110] direction to obtain the best thermoelectric performance.

This review is focused on the efforts to obtain stoichiometric nanowires with the proper orientation by electrodeposition and their thermoelectric properties for the V-VI thermoelectric compounds (Bi_2_Te_3_, Se-doped Bi-Te, Sb-doped Bi-Te, and SbTe). We will analyze the influence of the electrodeposition conditions on morphology, composition and thermoelectric properties.

## Electrodeposition of Bi_2_Te_3_ Nanowires

To obtain one-dimensional nanostructures, electrodeposition has some advantages over other growth methods as this technique enables obtaining high aspect-ratio structures, along with a good control over the crystallographic structure and the morphological properties. Moreover, it is cost effective and scalable to industrial requirements. In general, previously to the electrodeposition of nanowires, the electrodeposition of films was optimized, in order to find the most appropriate experimental parameters to obtain the desired composition, orientation, morphology, etc. In the literature, different reviews of electrodeposition thermoelectric films can be found (Xiao et al., 2008; Boulanger, 2010; Rostek et al., 2015). Therefore, in this work we will focus mainly on the electrodeposition of nanowires.

The most studied solution to obtain bismuth telluride films and nanowires is an aqueous solution of Bi^3+^, TeO^2+^, and HNO_3_. The mechanism of electrodeposition using this solution was investigated by Stacy group (Martín-González et al., 2002) in detail. Based on the Pourbaix diagram developed in that work, it can be seen the necessity for a strong acidic media to stabilize the ionic species in solution.

In the specific case of electrochemically grown nanowires, different templates can be used, which allow the production of high-density nanowire arrays and, at the same time, give them certain mechanical stability (Caballero-Calero and Martín-González, 2016). The two most common templates to obtain nanowires by electrodeposition are polycarbonate (PC) and anodic aluminum oxide (AAO) membranes, being both templates electrically insulating and robust.

Anodic aluminum oxide membranes (Masuda and Fukuda, 1995; Lee et al., 2006; Martín et al., 2012, 2013; Lee and Park, 2014; Manzano et al., 2014, 2016b; Sousa et al., 2014) exhibit a higher density of pores (up to 10^10^ pores/cm^2^) (Sander et al., 2003) and can achieve template thicknesses of more than 100 μm. These membranes display hexagonal ordering perpendicular to the surface and thermal conductivities of 1.07–1.32 W/m·K (Abad et al., 2016a). In the case of polycarbonate membranes, they have lower pore density (~10^8^ pores/cm^2^) (Koukharenko et al., 2008), so fewer nanowires can be fabricated at the same time. These templates present lower thermal conductivity than AAO (~0.2 W/m·K) (Picht et al., 2012). It is important to stay at this point that there is also the possibility of growing nanowires without the necessity of a template, with techniques such as decorating the step edge of a substrate, see for instance (Menke et al., 2004). However, from now on we will only review the state of the art in nanowires obtained by electrodeposition inside a template.

There are different electrodeposition modes: constant potential or current density, and pulsed electrodeposition line pulsing between two potentials, or two current densities, or by pulsing by a combination between potential and current density. The principal achievements in the fabrication of bismuth telluride nanowires grown by electrodeposition are collected in Table 1.

**Table 1:** Principal achievements in the fabrication of bismuth telluride nanowires grown by electrodeposition.

**Electrodeposition mode**	**Composition and orientation**	**Template**	**Nanowire diameter (nm)**	**References**
Constant current density	Bi_2_Te_3_ Polycrystalline	AAO	280 ± 30	Sapp et al., 1999
Constant potential	Bi_2_Te_3_ [110]	AAO	200, 75, 50, and 25	Prieto et al., 2001; Sander et al., 2002, 2003
Constant current density	Bi_2_Te_3_ [110]	AAO	50	Jin et al., 2004
Pulsed electrodeposition between two potentials	Bi_2_Te_3_ [015]	AAO	40-60	Liang et al., 2006
Pulsed electrodeposition between two potentials	Bi_2_Te_3_ [110]	AAO	40	Trahey et al., 2007
Constant potential, constant current density and pulsed electrodeposition between two potentials	Bi_2_Te_3_ [110]	AAO	50-80	Jongmin et al., 2008; Lee et al., 2010a,b; Peranio et al., 2012
Constant potential in DMSO	Bi_2_Te_3_ [015]	PC	60	Frantz et al., 2010, 2012
Pulsed electrodeposition in DMSO	Bi_1.55_Te_3.45_ [110]	AAO	70	Li W.-J et al., 2011
Constant potential	Bi_2_Te_3_ [110]	PC	15-25	Picht et al., 2012
Pulsed electrodeposition	Bi_2_Te_3_ [015]	AAO	15	Martín et al., 2013
Pulsed electrodeposition in P0CCOV	Bi_2_Te_3_ [110]	AAO	25,45,52, and 300	Muñoz Rojo et al., 2016, 2017; Rodríguez-Fernández et al., 2016
Pulsed electrodeposition in P0CCOV	3D-Bi_2_Te_3_ [110]	AAO	52	Martín et al., 2014; Ruiz-Clavijo et al., 2018

**Figure 2:**
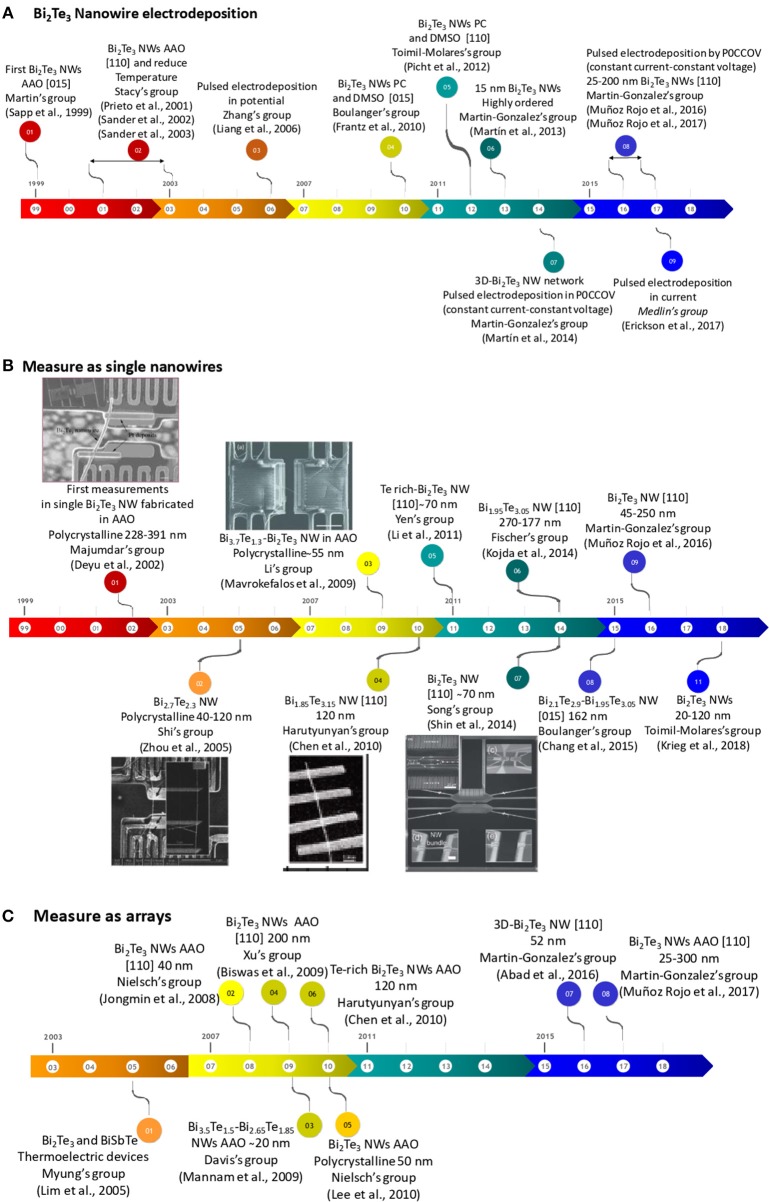
**(A)** Temporal line of the principal achievements in the fabrication of bismuth telluride nanowires grown by electrodeposition. **(B)** The temporal line of the thermoelectric measurements in single bismuth telluride nanowires grown by electrodeposition. **(C)** The temporal line of the thermoelectric measurements of bismuth telluride nanowires embedded inside templates grown by electrodeposition. Reproduced with permission from Deyu et al. (2002), Zhou et al. (2005), Mavrokefalos et al. (2009), Chen et al. (2010), and Shin et al. (2014). Copyright 2002 IEEE, 2005 Applied Physics Letters, 2009 Journal of Applied Physics, 2010 American Chemical Society and 2014 Royal Society of Chemistry.

The first work in electrodeposition of Bi_2_Te_3_ nanowires was reported in 1999 by Martin's group (Sapp et al., 1999) inside commercial anodic aluminum oxide. These nanowires were grown using constant current density in 280 ± 30 nm AAO templates. The resulting nanowires were polycrystalline. This landmark is the starting point of the temporal line for Bi_2_Te_3_ nanowire fabrication by electrodeposition that is presented in Figure 2.

From 2001 to 2003, Stacy's group spent many efforts in optimizing bismuth telluride nanowires. The obtained nanowires were oriented along [110] direction (which is the best direction to obtain the highest figure of merit for out-of-plane applications, with the *c-axis* perpendicular to the length of the nanowires, as explained before), a high filling ratio (~80%) and a high aspect ratio (~1,000) for diameters of 200, 75, 50, and 25 nm (Prieto et al., 2001; Sander et al., 2002, 2003). The electrochemistry was performed at 2°C.

Later on, Li's group worked on the growth of bismuth telluride nanowires also oriented along [110] direction, with a high filling ratio, uniform growth and 50 nm in diameter, by applying constant current density (Jin et al., 2004). In 2006 and 2007, different studies were reported changing the different parameters involved in the electrochemical process: using different reduction potentials to change the composition and crystallographic orientation (Wang et al., 2006), different Te and Bi concentrations in the electrolyte (Jun et al., 2006) and thermal annealings performed after the growth of the nanowires (Kim et al., 2007).

In order to further improve the filling factor of the nanowires, pulsed electrodeposition has been proved to be the best solution. The most commonly used method, so far, is pulsing between two different potentials at room temperature. The deposition consists on applying the reduction potential during a certain time, *t*_*on*_, and then introduce a “rest” potential during a certain time, *t*_*off*_. In 2006, Zhang's group (Liang et al., 2006) published the first study based on Bi_2_Te_3_ nanowires grown by pulsed-potential deposition. In this work, the effect of the reduction potential and the duration of the pulses in the crystallographic structure of the nanowires was analyzed. The produced nanowires were all oriented along the [015] direction. One year later, Stacy's group (Trahey et al., 2007) reported a study based on Bi_2_Te_3_ nanowires grown by pulsed-potential deposition in order to improve the filling ratio by enhancing the nucleation of the nanowires, which showed also the [110] direction. The nucleation rate obtained was found to be 95%. Furthermore, it was concluded that reducing the bath temperature to temperatures lower than 4°C (as they were doing in the previous works) improved the alumina filling factor and the nanowires crystallinity. In contrast, nanowires that were grown at temperatures between 7 and 10°C or around room temperature (22 to 23°C) presented more crystallographic orientations. In 2008, Nielsch's group (Jongmin et al., 2008) published a study where bismuth telluride nanowires grown at different deposition modes (constant potential, constant current density, and pulsing between two potentials) were compared. Pulsed-potential deposition showed nanowires preferably oriented along [110] direction. This mode was the procedure used by Nielsch's group from 2010 to 2012 (Lee et al., 2010a,b; Peranio et al., 2012). The nanowires reported in these papers followed Lee's et al. (Jongmin et al., 2008) recipe. Those nanowires presented a uniform filling ratio with 50–80 nm in diameter and 25–60 μm in length and were preferentially oriented along [110] direction.

Between 2010 and 2012, Boulanger's group (Frantz et al., 2010) reported for the first time the electrodeposition of Bi_2_Te_3_ nanowires in DMSO using PC membranes. They used a mixture of dimethyl sulfoxide (DMSO) and water. In the presence of DMSO, the current density decreases by a factor four in comparison with an aqueous solution. Because of this reduction in the diffusion coefficient, the growth rate of the nanowires is lower. This improves the filling ratio of the templates. Also a reduction in their crystallite size was observed when compared to those grown in aqueous solution (Frantz et al., 2010). The nanowires obtained in these studies were 60 nm in diameter, polycrystalline, and 25–30 μm in length, with a preferential orientation along [015] direction (Frantz et al., 2010, 2012) and homogeneous in composition (reaching the stoichiometric Bi_2_Te_3_) along the length of the nanowires (Frantz et al., 2012). In 2011, pulsed-potential electrodeposition was studied by Yen's group using a DMSO solution, and those nanowires exhibited a uniform filling ratio with 70 nm in diameter and 41 μm in length (Li W.-J et al., 2011). The nanowires were preferentially oriented along [110] direction and they had a composition of Bi_1.55_Te_3.45_, a little bit far from the stoichiometry.

In 2012, Toimil-Molares's group (Picht et al., 2012) observed a change in the crystallographic orientation by adjusting the reduction potentials, bath temperature (from 20 to 4°C), and electrolyte concentration for nanowires grown in ion-track PC membranes. Also, in 2012, Outzourhit's group (Elyaagoubi et al., 2017) reported the electrodeposition of Bi_2_Te_3_ nanowires inside 52 nm pore AAO templates grown with the DMSO:H_2_O solution (Frantz et al., 2010). The nanowires obtained were polycrystalline and with a small crystallite size of 30 nm.

**Figure 3:**
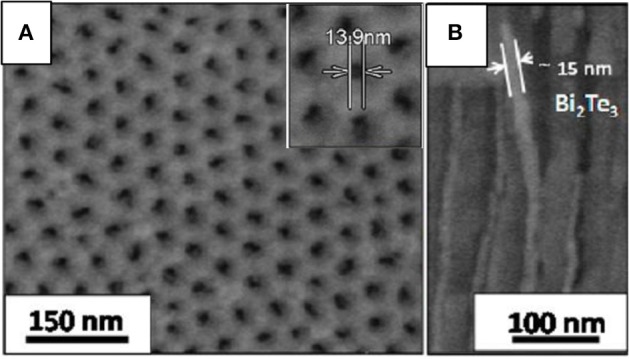
**(A)** SEM images of highly ordered 14 nm pore diameter AAO templates and **(B)** a cross-section by SEM of 15 nm Bi_2_Te_3_ nanowires inside the AAO nanopores. Adapted with permission from Martín et al. (2013). Copyright 2013 ACS Publications.

In 2013, Martin-Gonzalez's group reported 15 nm in diameter Bi_2_Te_3_ nanowires in AAO membranes with [015] as the main diffraction maxima (Martín et al., 2013). In order to do that, the group developed porous alumina templates with 15–12 nm in diameter by the addition of ethylene glycol during aluminum anodization (Martín et al., 2013; Manzano et al., 2014). And, more recently, the group has extended the process to obtain sub-10 nm templates (Resende and Martín-González, 2019) in concentrated sulphuric acid and 25% _V/V_ of ethanol anodized under low current densities. Figures 3A,B show SEM images of 14 nm pore diameter AAO templates and 15 nm in diameter Bi_2_Te_3_ nanowires embedded into the AAO templates, respectively.

In 2014, Song's group generated 55 nm nanowires in AAO templates oriented in the [110] direction using constant potential in standard aqueous solution. They concluded that changing the reduction potential, the number of twins inside the nanowires (and so the crystallite size) could be controlled (Shin et al., 2014). In the same year, Fischer's group reported a similar deposition method, pulsed-potential, to obtain nanowires with 190–320 nm in diameter and oriented preferentially along [110] direction (Kojda et al., 2014). From 2014 to 2016, Bi_2_Te_3_ nanowires with different compositions were obtained applying different reduction potentials (Ng et al., 2014, 2016; Kok et al., 2016; Proenca et al., 2017). In 2015, Nandhakumar's group reported nanowires with 80 nm in diameter, obtained using aqueous solution into PC membranes with [110] as preferential direction (Koukharenko et al., 2015). In 2016, Cantarero's group reported Raman measurements performed in Bi_2_Te_3_ nanowires grown in AAO templates by Martin–Gonzalez's group (Rodríguez-Fernández et al., 2016). In this study, nanowires with different compositions were fabricated changing the reduction potential on purpose. The conclusion of the study is that the Raman spectra of Te-rich bismuth telluride nanowires exhibited an additional peak compared to stoichiometric Bi_2_Te_3_ nanowires. This peak corresponded to Te nanocrystals that cannot be observed by XRD because they are very small. Therefore, Raman spectroscopy seems to be a perfect tool to detect Te clusters inside the nanowires.

In 2016, Martin-Gonzalez's group published the first nanowires produced by pulsing between a certain potential and zero current. This procedure was tested in films before, see for example (Manzano et al., 2013). This methodology was used for the first time to obtain stoichiometric bismuth telluride films highly oriented along [110] direction (Manzano et al., 2013). This procedure has been called Pulsed ZERO Current COnstant Voltage (P0CCOV). It consists in alternating between potentiostatic and galvanostatic modes during each pulse, instead of pulsing between two potentials—only potentiostatic— (as done until that moment for Bi_2_Te_3_) or pulsing between two currents—only galvanostatic-, which is the other conventional way of pulsing. The reason to choose that different option of pulsing is that during the *on* time, the ions are deposited on the electrode surface and their concentration decreases at the interface between the substrate and the electrolyte. By using zero current periods during the *off* time, the system is allowed to be in a truly resting state, which helps to redistribute the remaining ions at the interface. In other words, at current = 0 A, there is no current going through the interface electrode/solution, so no driving force is applied during that time and the system can truly rest. This is slightly different than pulsing between one potential and the open circuit potential (OCP). The OCP was calculated at the beginning of the process, however the electrode surface is not the same as deposition takes place. The surface of the electrode changes in each pulse. Therefore, the initial OCP is not a real OCP after each pulse and at the initial OCP value, a residual current is still going through the system. While in the P0CCOV procedure the current is always 0 during the off time. So, no current is passing, allowing a real resting time for the electrodeposit material.

**Figure 4:**
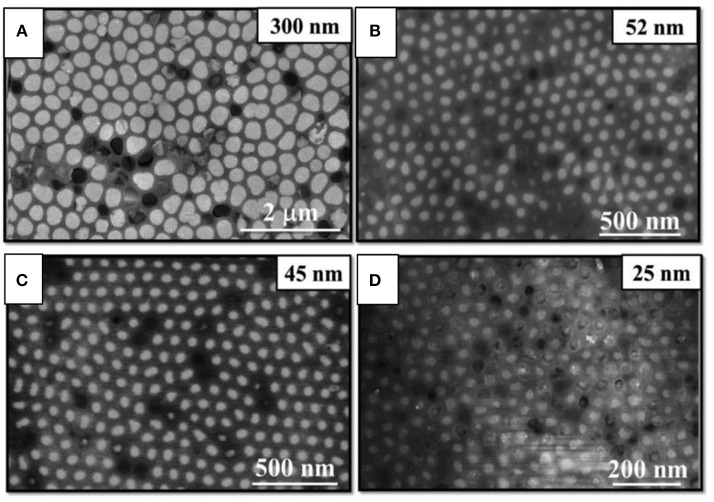
SEM images of bismuth telluride nanowires with average different diameters: **(A)** 300 nm, **(B)** 52 nm, **(C)** 45 nm, and **(D)** 25 nm. Reproduced with permission from Muñoz Rojo et al. (2017). Copyright 2017 Nanoscale.

By this P0CCOV procedure bismuth telluride nanowires with different pore diameters (300, 52, 45, and 25 nm), high filling ratio, oriented along the [110] direction and with the desired stoichiometry (Bi_2_Te_3_) were obtained at 0°C (Muñoz Rojo et al., 2016, 2017). The length of these nanowires were 32, 50, 42, and 25 μm for different diameters 300, 52, 45, and 25 nm, respectively. Figure 4 shows SEM images of the top view of Bi_2_Te_3_ nanowires for the different diameters.

In 2017, Medlin's group (Erickson et al., 2017) reported the electrodeposition at pulsed galvanostatic mode between two different current densities, followed by thermal annealing between 150 and 350°C to improve the crystallinity of stoichiometric Bi_2_Te_3_ nanowires with 75 nm in diameter. Those nanowires were oriented preferentially along [110] direction. The same year, bismuth-rich bismuth telluride nanowires with different diameters (10–275 nm) were electrodeposited using different current densities in order to change the composition of the wires by Hill's group. In order to reduce the pore diameter of the AAO templates, silica was deposited by dip-coating before the electrodeposition (Ryan et al., 2017).

It is known that the use of additives reduces the roughness of electrodeposited films. Normally, additives such as ethylenediaminetetraacetic acid (EDTA) are used because it forms a complex with bismuth and thus reduces its diffusion coefficient, or sodium lignosulfonate (SLS) (Kuleshova et al., 2010; Naylor et al., 2012; Caballero-Calero et al., 2014; Abad et al., 2015) which is used in the electrodeposition of tellurium and selenium because it reduces the crystallite size; this additive allows the formation of films with less roughness and favors the [110] orientation. Furthermore, White's and Martin-Gonzalez's groups observed an improvement in the Seebeck coefficient of Bi_2_Te_3_ films (Kuleshova et al., 2010; Caballero-Calero et al., 2014) and Se doped-BiTe films (Caballero-Calero et al., 2018) with the use of lignosulfonates like sodium lignosulfonate (SLS) in the solution. Nevertheless, the effect of additives is still to be studied in nanowires.

In conclusion, the most relevant results over the last two decades are that the smallest stoichiometric Bi_2_Te_3_ nanowires achieved are 15 nm in diameter. There are available stoichiometric nanowires oriented along the [110] direction in different diameters to perform different measurements. Pulsed electrodeposition (in its different forms) and bath temperatures close to 0°C seem to be the best solution to improve the filling factor of the templates and to have high aspect-ratio (≈1,000).

## Thermoelectric Properties

As we have seen in the previous section, extensive work has been done in order to obtain stoichiometric nanowires, highly oriented along [110], and with different diameters. Now in this section, we are going to discuss the different measurements that have been performed to study the influence of the diameter in the different properties that affect their thermoelectric performance, such as Seebeck coefficient, thermal conductivity, and electrical conductivity.

In general, we can say that there are two approaches to measure the thermoelectric properties of bismuth telluride nanowires. One starts by selectively dissolving the template, dispersing or placing the nanowires in a microchip, and measuring the performance of a single nanowire. The second approach consists of carrying out the measurements on the nanowires embedded inside the templates for both polycarbonate membranes and anodic aluminum oxide matrices.

### Thermoelectric Measurements on Single Nanowires

**Figure 5:**
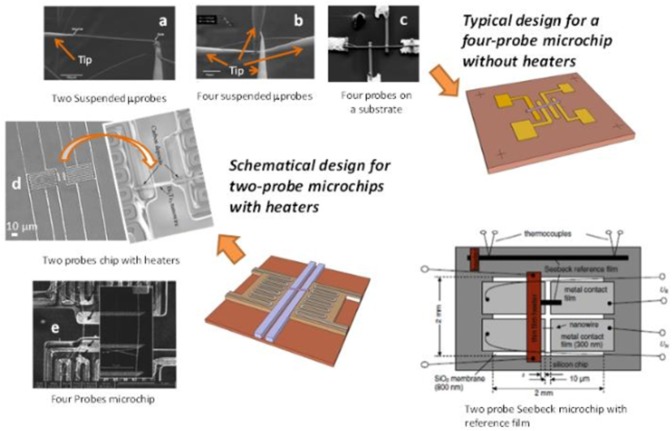
Some types of microchips used for transport properties measurements in the literature. Reproduced with permission from Rojo et al. (2013). Copyright 2013 Nanoscale. **(a)** Two suspended μprobes, **(b)** four suspended μprobes, **(c)** four probes on a substrate, **(d)** two probes chip with heaters and **(e)** two probes microchip.

After selectively dissolving the template, the nanowires are obtained free-standing normally in solution. This procedure sometimes generates an oxide layer on the surface of the nanowire. Then, they must be placed in the right position on top of a lab fabricated microchip and contacts must be done to assure good electrical and thermal conductivity. Once all of that is done, different measurements can be performed, depending on the microchips configuration. For a review of the different type of microchips, please take a look to Rojo et al. (2013). Some of those kinds of microchips can be found in Figure 5.

In 2002, Majumdar's group (Deyu et al., 2002) reported for the first time the thermal conductivity (2.2–0.13 W/m·K for 228 and 391 nm, respectively) and Seebeck coefficient (-50 μV/K for 391 nm) of bismuth telluride nanowires grown in AAO by Stacy's group. The chip used for these measurements was a suspended two probes microfabricated chip with heaters. In that work, different contacts were tested.

In 2005, Shi's group (Zhou et al., 2005) reported thermoelectric measurements (σ, *S*, and κ) in single bismuth telluride nanowires grown in AAO. The nanowires were oriented along [110] direction with different compositions of bismuth and tellurium. Depending on the composition, different σ, *S*, and κ were obtained. The electrical conductivity, Seebeck coefficient and thermal conductivity at 300 K were found to be 0.8·10^3^-2·10^3^ S/cm, −10 – −35 μV/K and 0.8–1.6 W/m·K. In this case, the composition of the nanowires were not stoichiometric. They were Bi-rich or Te-rich. The measurements were performed using four probes microchips.

In 2009, Li's group (Mavrokefalos et al., 2009) reported the thermoelectric properties (σ*, S*, and κ) of single bismuth telluride nanowires in AAO membranes measured using a four probes microchip. Here, the novelty was the use of thermal annealing in forming gas to make contact between the nanowires and the pre-patterned electrode to perform the measurements in order to reduce the superficial oxide layer. Polycrystalline nanowires with different compositions (Bi_2.15_Te_2.85_, Bi_3.7_Te_1.3_, and Bi_2_Te_3_) were measured. Seebeck coefficients of −65 and −45 μV/K were obtained for Bi-rich Bi_3.7_Te_1.3_ and Bi_2_Te_3_, respectively at 300 K. The electrical conductivities range between 500 and 2200 S/cm at 300 K for Bi-rich Bi_3.7_Te_1.3_ and Bi_2_Te_3_, respectively. In addition, the thermal conductivities varied from 1.4 to 2.8 W/m·K for Bi-rich Bi_3.7_Te_1.3_ and Bi_2_Te_3_, respectively. In conclusion, the maximum figure of merit *zT* was found to be 0.06 for stoichiometric Bi_2_Te_3_ nanowires at RT and that value increases to *zT* = 0.22 at ~450 K. The main conclusion is that better control over chemical composition is necessary to improve the *zT* of the electrodeposited NWs. In particular, they propose to maximize the care to minimize impurities present in the electrochemical deposition setup to avoid unintentional doping of the NWs.

One year after, Harutyunyan's group (Chen et al., 2010) reported the electrical resistivity of a single Te-rich Bi_1, 85_Te_3, 15_ nanowire grown at constant potential in aqueous solution using commercial AAO template. The nanowires were preferentially oriented along [110] direction with 120 nm in diameter. The electrical resistivity was found to be 12–4.3 μΩ·m for 1 to 350 K. The electrical resistivity decreased exponentially when the temperature increased. This value is smaller than the value expected for bulk Bi_2_Te_3_, 19 μΩ·m (Fleurial et al., 1988), probably due to the excess of tellurium of the nanowires. In general, when an excess of tellurium is observed, the electrical conductivity is much lower and the Seebeck coefficient is slightly higher than in stoichiometric Bi_2_Te_3_, which explains the results obtained. In this case, the measurements were performed using four probes microchips.

In 2011, Yen's group (Li W.-J et al., 2011) measured a value of 105 μΩ·m for Te-rich Bi_2_Te_3_ nanowires oriented along [110] direction, with 60–70 nm in diameter and grown in a DMSO solution by potential pulsed deposition inside AAO templates. According to the authors, this value is one order of magnitude higher than the value reported for single crystal Te-rich Bismuth Telluride (2.6 μΩ·m) (Rowe, 1995). The chip used in this case was a four-probes microchip.

In 2014, Song's group (Shin et al., 2014) reported the electrical conductivity of twin-free and twin-containing Bi_2_Te_3_ nanowires oriented preferentially along [110] direction and with 60–70 nm in diameter grown in AAO. The electrical conductivity value was similar for both cases, with a value of 2.3·10^5^ S/m. However, the Seebeck coefficient was slightly smaller in the case of twin-containing (~57 μV/K) than in the case of twin-free (~70 μV/K). The thermal conductivity was smaller in the case of twin-containing (1.9 W/m·K) than in twin-free (2.3 W/m·K) due to the reduction of the carrier concentration and phonon scattering (Shin et al., 2014). The chip used in this case was a four suspended microprobe chip. At the same time, Fischer's group published a study of Bi_1.95_Te_3.05_ nanowires grown in AAO using a two probes chip with heaters. The nanowires were oriented preferentially along [110] direction grown by pulsed electrodeposition with an electrical conductivity of 1.3–2.3·10^5^ S/m, a Seebeck coefficient of −41 – −45 μV/K and thermal conductivity of 0.9–2 W/m·K for 270 and 177 nm in diameter, respectively (Kojda et al., 2014). This variation was explained by the difference in diameter along the nanowire.

In 2015, Boulanger's group reported the electrical conductivity and Seebeck coefficient of nanowires grown in PC membranes using DMSO solution. The nanowires were oriented along [015] direction with 162 nm in diameter, the value of the electrical conductivity and Seebeck coefficient was found to be 0.1–4·10^4^ S/m and −10 – −80 μV/K for different concentrations of bismuth and tellurium, 0.42 and 0.39, respectively (Chang et al., 2015). In this case, the measurements were performed using four probes microchips.

In 2016, Martin-Gonzalez's group published a paper of stoichiometric Bi_2_Te_3_ nanowires grown in AAO templates. The nanowires were oriented preferentially along [110] direction grown by pulsed electrodeposition where the electrical conductivity was found to be 1·10^4^, 1.6·10^4^ and 2.9·10^4^ S/m for different nanowire diameters, 250, 70, and 45 nm, respectively. The measurements were done using a four suspended microprobes chip. Furthermore, in this paper, the topological character of bismuth telluride nanowires was shown and the significance of the topological insulator surface states in room temperature nanowire working devices was demonstrated (Muñoz Rojo et al., 2016), since the ripples did not disappear when an electric current was passing through the nanowire.

More recently, 2018, Toimil-Molares's group reported an increment in the electrical resistivity from 0.2 to 2.4 mΩ·cm for diameters from 120 to 20 nm in Bi_2_Te_3_ nanowires oriented preferentially along [110] and [205] direction using polycarbonate membranes (Krieg et al., 2018). Furthermore, magneto-transport properties of these single nanowires were reported in this study. A metallic behavior was observed due to the highly-degenerate nature of the nanowires. The reduction in the electrical resistivity as diameter increases confirmed the quasi-ballistic nature of charge carriers, with the reduction of the number of surface conductance channels in disordered quantum wires with stronger quantum confinement. The chips used in this case were four probes microchips. The summary of the thermoelectric measurements of individual bismuth telluride nanowires is collected in Table 2 in white.

**Table 2:** Thermoelectric measurements of individual Bi_1−x_Te_x_ nanowires found in the literature.

**Composition and orientation**	**Nanowire diameter (nm)**	**Template**	**σ (S/cm)**	**S (μV/K)**	**PF (μW/K^2^·m)**	**k (W/m·K)**	**References**
**NON-STOICHIOMETRIC NANOWIRES**							
Bi-rich Bi_7_Te_5_-Bi_4_Te_5_	10–40	AAO	–	2–79	–	2.4–0.1^#^	Ryan et al., 2017
Bi_2.7_Te_2.3_ [110]^*^	40–120	AAO	0.8·10^3^–2·10^3^	−10– (−35)	8–2.5 10^2^	0.8–1.6	Zhou et al., 2005
Bi-rich and Te-rich Bi_3.5_Te_1.5_-Bi_2.65_Te_1.85_	~20	AAO	0.33–25+	−318.7–117	3.4–3.4 10^1^	–	Mannam et al., 2009
Bi_2_Te_3_ Bi_2.15_Te_2.85_ Bi_3.7_${\text{Te}}_{1.3}^{*}$ Polycrystalline	~55	AAO	5·10^2^–2.2·10^3^	−45– (−65)	1.0·10^2^–9.3·10^2^	1.4–2.8	Mavrokefalos et al., 2009
Bi-rich Bi_2.1_Te_2.9_ Te-rich Bi_1.95_Te_3.05_ [015]	162	PC	0.1·10^2^–4·10^2^	−10–(−80)	1.96·10^2^	–	Chang et al., 2015
Bi_2_Te_3_ Polycrystalline^*^	228–391	AAO	–	−50 (391 nm)	–	2.2–0.13	Deyu et al., 2002
Te-rich Bi_2_Te_3_ Polycrystalline	60–70	AAO	9.5·10^1^	–	–	–	Li W.-J et al., 2011
**NEAR TO STOICHIOMETRIC NANOWIRES**							
Bi_1.9_Te_3.1_ Preferential [110]	120	AAO	8.3·10^2^–2.3·10^3^	–	–	–	Chen et al., 2010
Bi_1.9_Te_3.1_ Preferential [110]	120	AAO	–	−70	–	0.75+	Chen et al., 2010
Bi_1.95_Te_3.05_[110]	177–270	AAO	2.3·10^3^–1.3·10^3^	−45– (−41)	4.7·10^2^–2.2·10^2^	2–0.9	Kojda et al., 2014
**STOICHIOMETRIC NANOWIRES**							
Bi_2_Te_3_ [110]	25–300	AAO	–	–	–	0.52–1.78^#^	Muñoz Rojo et al., 2017
Bi_2_Te_3_ [110] preferential orientation	40	AAO	10^3^+,?	−30	9·10^2^		Jongmin et al., 2008
Bi_2_Te_3_[110]	45–250	AAO	2.9·10^2^−−10^2^	–	–	–	Muñoz Rojo et al., 2016
Bi_2_Te_3_[110] Twins and without twins	60–70	AAO	2.3·10^3^	~−57– ~(−70)	5 ·10^2^–7.5·10^2^	1.9–2.3	Shin et al., 2014
Bi_2_Te_3_ [110]	200	SU-8	–	–	–	1.44+	Biswas et al., 2009
Bi_2_Te_3_ [110]	200–400	AAO	–	–	–	1.37^#^	Muñoz Rojo et al., 2013
Bi_2_Te_3_	50	AAO	16.9·10^2^ +	−55	4.7·10^2^	–	Lee et al., 2010b
Bi_2_Te_3_[110] and [205]	20–120	PC	5·10^3^–4.2·10^2^	–	–	–	Krieg et al., 2018

In white are the measurements performed in single nanowires using microchips. In light green are the measurements performed in the nanowires embedded in a template. “^*^” Stands for manuscripts were several NWs compositions were measured in microchips. “+” Stands for direct measurements within templates, “^#^” corresponds to calculated values using the effective medium theory, The symbol “?” corresponds to values that the authors are not sure about.

### Thermoelectric Measurements of Nanowires Embedded Inside the Template

**Figure 6:**
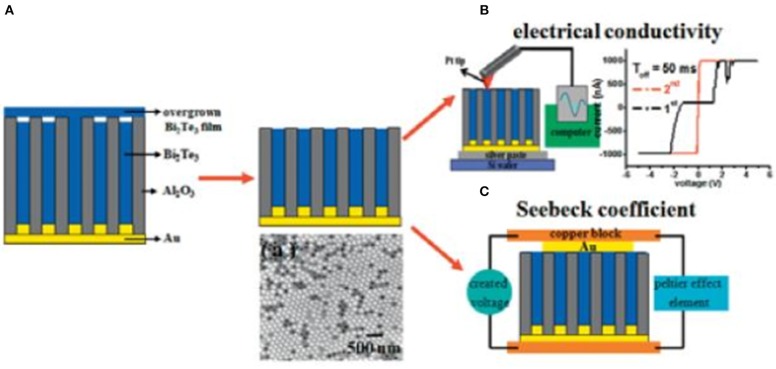
**(A)** Scheme diagram of the process to prepare the nanowires for thermoelectric measurements. The initial overgrown at the top of the template is polished as shown in this figure. A SEM top view of the nanowires is shown. **(B)** The electrical conductivity setup for individual nanowire and **(C)** Seebeck coefficient of nanowire arrays. Reproduced with permission from Lee et al. (2010b). Copyright 2010 Physica Status Solidi (RRL).

For these measurements, the nanowires are not extracted from the template. So the oxide layer that appears upon template selective etching is avoided. Nanowire arrays embedded in matrices have a big advantage, given that it is possible to integrate the material directly into real thermoelectric devices (Biswas et al., 2009). Another interest of measuring the thermoelectric properties in nanowires embedded inside the templates is that the output current is enhanced (more nanowires are being measured at the same time, not only one) and the template adds mechanical stability to the system. There are different techniques to measure the nanowires embedded inside the templates (Rojo et al., 2013; Abad et al., 2017). For example, to measure the electrical conductivity one can use an AFM with a conductive tip (Muñoz Rojo et al., 2016). To measure the Seebeck coefficient, a development that uses two blocks of Cu has been used in several works, as it can be seen in Figure 6. For thermal conductivity, the most commonly used techniques are laser flash, 3*w*-scanning thermal microscopy (Muñoz Rojo et al., 2013), and Photoacoustic method (Muñoz Rojo et al., 2017). In Figure 6A, a diagram of the thermoelectric properties of nanowires embedded inside the templates is shown, with two examples of actual measurements (AFM conductive tip and two copper blocks).

Historically, the first measurements of this kind appeared in 2004, when Chen's group (Borca-Tasciuc et al., 2004) reported the thermal diffusivity (6.9·10^−7^ m^2^/s at 300K) of bismuth telluride nanowires embedded into an AAO template grown by Stacy's group. Later, in 2008, Nielsch's group (Jongmin et al., 2008) published the electrical resistance (10^−5^ Ω·m) and Seebeck coefficient (−30 μV/K) of bismuth telluride nanowires grown via pulsed-potential deposition, being these nanowires highly preferred oriented along [110] direction with 40 nm in diameter. One year later, the electrical resistivity (30 and 0.4 mΩ·m) and Seebeck coefficient (−318.7 and 117 μV/K) for n and p-type non-stoichiometric bismuth telluride nanowires (Bi_3.5_Te_1.5_ and Bi_2.65_Te_1.85_), were presented by Mannam et al. (Mannam et al., 2009).

In 2009, the thermal conductivity of stoichiometric Bi_2_Te_3_ nanowires embedded in 200 nm AAO templates was published by Xu's group (Biswas et al., 2009). To perform the measurements, the AAO membrane (κ = 1.31 W/m·K) was replaced by an epoxy (SU-8) with lower thermal conductivity (0.2 W/m·K). The thermal conductivity of the nanowires measured was 1.44 W/m·K. It is worth noting that this value is smaller than the value found for single crystal bulk stoichiometric bismuth telluride (2.2 W/m·K) when the crystal structure was oriented perpendicular to the *c-axis* (Jacquot et al., 2010).

In 2010, Nielsch's group (Lee et al., 2010b) reported the electrical conductivity and Seebeck coefficient of stoichiometric Bi_2_Te_3_ nanowires inside AAO templates grown by pulsed-potential deposition. The nanowires were oriented preferentially along [110] direction with 50 nm in diameter. The electrical conductivity was measured using a conductive AFM tip and Seebeck coefficient was obtained placing the sample in an experimental system that consist of two Cu blocks (shown in Figure 6C). The electrical conductivity and Seebeck coefficient were found to be 5.3–16.9·10^4^ Ω^−1^·m^−1^ and 46.6–55 μV/K, respectively, for polycrystalline Bi_2_Te_3_ nanowires oriented preferentially along [110] direction (Lee et al., 2010b). The Seebeck coefficient measured by Harutyunyan's group (Chen et al., 2010) using the same method in Te-rich bismuth telluride nanowires with 120 nm in diameter grown by constant potential was −70 μV/K. The thermal conductivity for these nanowires was 0.75 W/m·K, being this parameter measured using a laser flash system.

In 2013, Martin-Gonzalez's group (Muñoz Rojo et al., 2013) reported the thermal conductivity, measured by 3*w*-scanning thermal microscopy, for Bi_2_Te_3_ nanowires preferentially oriented [110] and with 200–400 nm in diameter grown at a constant potential, obtaining a value of 1.37 W/m·K.

In 2017, the conductivity ratio of electrical-to-thermal and the Seebeck coefficient were measured using a modified Harman technique (Ryan et al., 2017) in Bi-rich bismuth telluride nanowires. A decrease in the electrical-thermal conductivity ratio by the Lorenz number, *L*_*o*_*T* at 400 K, (0.24–0.08) was measured as nanowire diameter increased (10–40 nm) and the Seebeck coefficient was found to be two orders of magnitude lower than that found in the literature (Jongmin et al., 2008; Mannam et al., 2009) and it did not correlate with the diameter. This variation in the results can be due to the difficulty of using Harman methods to measure nanowires. For example, a theoretical study using COMSOL simulations for thermoelectric measurements employing Harman technique in films and nanowires, concluded that there is a strong dependence of *f*_*HF*_ on nanowire diameter, resulting in very high frequencies needed for the measurments (*f*_*HF*_ > 106 Hz for diameters below 250 nm). Therefore, this method cannot be applied for nanowires with diameter below 25 nm (Munoz Rojo et al., 2015).

In the same year, Martin-Gonzalez's group (Muñoz Rojo et al., 2017) reported a decrease of 70% in the thermal conductivity (from 1.78 to 0.52 W/m·K) when the nanowire diameter decreases (300 to 25 nm) using the photoacoustic and the SThM methods (Rojo et al., 2013; Wilson et al., 2015; Abad et al., 2017), and it was validated by theoretical calculations using the Kinetic-Collective model. The reduction in the thermal conductivity of the nanowires can be explained in terms of an increment of phonon scattering. The main conclusion of that work is that the smaller the diameter of the nanowires, the larger the modification in the mean free path of the low-frequency phonons. These Bi_2_Te_3_ nanowires were grown by pulsed electrodeposition and were oriented preferentially along [110] direction. The summary of the thermoelectric measurements of bismuth telluride nanowires embedded into the template is collected in Table 1 in light green/gray.

### Discussion of the Different Results Obtained in Thermoelectric Nanowires

In general, from the results of Table 2, it can be concluded that as nanowire diameter decreases, electrical conductivity seems to increase slightly when compared with similar electrodeposited films. This conclusion can be reached when taking into account only the studies performed in stoichiometric Bi_2_Te_3_ nanowires oriented along [110] direction using PC and AAO templates. Those publications where, either the stoichiometry of the nanowires is not 2:3, or the orientation is not [110] have not been taken into account because the results obtained in such studies are not comparable, given that the different thermoelectric properties also depend on the stoichiometry and on the orientation of the nanowires.

**Figure 7:**
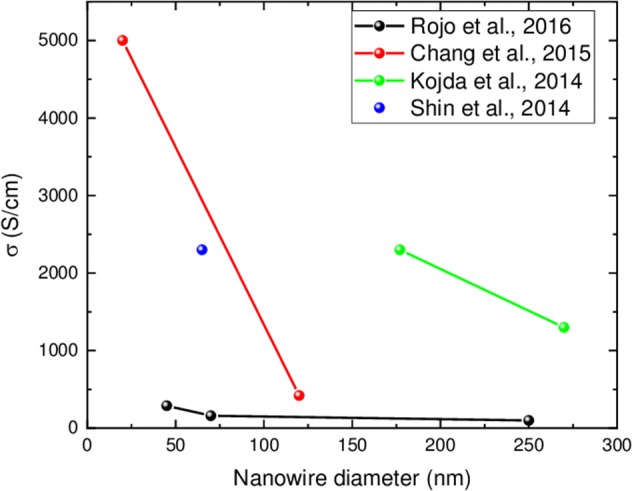
Electrical conductivity as a function of nanowire diameter extracted from different works. They have been measured using different techniques. The values shown are at room temperature. Only data for stoichiometric Bi_2_Te_3_ nanowires oriented along [110] direction are compiled in this graph.

Figure 7 shows the electrical conductivity as a function of nanowire diameter from different publications, where stoichiometric Bi_2_Te_3_ nanowires oriented along [110] direction have been measured at room temperature. In this graph, only the studies where the authors show in a reliable way that the nanowires are stoichiometric and oriented along [110] direction are collected. In more detail, the electrical conductivity increases from 4.2·10^2^ to 5·10^3^ S/cm for 120 to 20 nm nanowires diameter, respectively (Chang et al., 2015); or from 10^2^ to 2.9·10^2^ S/cm for 250 to 45 nm nanowires diameter, respectively (Muñoz Rojo et al., 2016); or from 1.3·10^3^ to 2.3·10^3^ S/cm for 270 to 177 nm nanowires diameter, respectively (Kojda et al., 2014).

One possible explanation for the increase in electrical conductivity upon nanowire diameter reduction can be due to the presence of the surface states since, as it was mentioned before, bismuth telluride nanowires are topological insulators (Gooth et al., 2015; Muñoz Rojo et al., 2016). Although, these topological states must be further studied by theoretical models to better comprehend the underlying physics, their presence in all the stoichiometric Bi_2_Te_3_ nanowires will help to understand the increment of the electrical conductivity. Nevertheless, other explications are also plausible.

The variation in the electrical conductivity between the different works shown in Figure 7 could be also explained taking into account the lack of metrology to calibrate the systems at the nanoscale. Another possible reason, when using microchips to measure single nanowires, can be the presence of an oxide shell in the outer part of the nanowires that is formed upon dissolving the template and by air exposure. In these measurements, another source of error can be the different type of electrical and thermal contacts used in the different studies. There is a wide variety of ways to contact the nanowires some examples are using Pt-carbon, 10 nm (Kojda et al., 2014) Ni/100 nm Al (Shin et al., 2014), 5 nm Cr/250 nm Au (Chang et al., 2015), or Au/tungsten (Muñoz Rojo et al., 2016). This contact resistance has a strong influence on the final values. Other possible reason can be the different sources of the reagents used to grow the nanowires. For example, the tellurium sources have been shown to have an important effect on the electrical conductivity in stoichiometric Bi_2_Te_3_ films (Manzano et al., 2018), since different companies have different impurities which can dope the semiconductor. The different tellurium sources used have been Te 99.999% from Fluka (Kojda et al., 2014), TeO_2_ (the purity was not specified) from Across organic (Shin et al., 2014), Te 99.7% from Prolabo (Chang et al., 2015) and Te 99.99% from Sigma Aldrich (Muñoz Rojo et al., 2016). All of these sources have different types of impurities that affect the final conductivity of the semiconductor, no matter the purity of the precursors (Manzano et al., 2018).

With respect to the Seebeck coefficient (see Table 2), maintaining the focus on stoichiometric Bi_2_Te_3_ nanowires with the appropriate orientation, there is not a clear correlation between the diameter and the Seebeck coefficient in the literature so far. The accurate measurements of the thermal gradient applied to the nanowires (which is not an easy task) and the recorded electrical voltage can be greatly affected by the way of measuring and usually the error is not reported. Then, in a very general way, it is found that the Seebeck coefficient in bismuth telluride nanowires is of the same order of magnitude in all the cases. For instance, in the largest diameters, 177 nm and 270 nm, a Seebeck coefficient of −45 and −41 μV/K was measured, respectively (Kojda et al., 2014). For smaller diameter nanowires of 50 nm, the Seebeck coefficient was measured, −55 μV/K (Lee et al., 2010b). By comparing those measurements one can think that there is a slight increase in the Seebeck upon size reduction. But the only measurement found of the Seebeck coefficient for nanowires of 40 nm in diameter (the smallest mentioned in the literature for Seebeck coefficient measurements) was found to be −30 μV/K (Jongmin et al., 2008), which is also in the same order of magnitude as the previous ones. Moreover, a value of −70 μV/K, for nanowires of 60–70 nm in diameter was measured, which is slightly higher than the previous values reported for nanowires. In another work, a reduction in the Seebeck coefficient (from −70 to −57 μV/K) was observed when the number of twins in the structure was increased (Shin et al., 2014). The use of different sources of the reagents that can dope the semiconductor, as demonstrated for bismuth telluride films (Manzano et al., 2018) affects the doping level and, thus, the Seebeck coefficient. This type of discrepancy in the Seebeck coefficient is also found in literature for electrodeposited Bi_2_Te_3_ films oriented along [110] direction, as it can be seen for example in the Seebeck coefficient measured for equivalent electrodeposited films, where the authors found a value of −40 μV/K in one case (Fleurial et al., 1999) and −70 μV/K in another work (Li et al., 2008). In any case, one should not forget the lack of metrology at the nanoscale, as another source of discrepancy between different works, as already mentioned before. Then, it can be concluded that the Seebeck coefficient values observed are within the same order of magnitude and no clear trend with the nanowire diameter has been found. Actually, taking into account the theory that predicted an increase of the Seebeck coefficient upon decreasing the dimensionality of the thermoelectric materials, it would be necessary to reach smaller diameters to see the effect of nanostructuration in the case of Bi_2_Te_3_. For the future, it will be interesting to have a work in which different diameters with stoichiometric nanowires are fabricated using the same reactants and measured in the same kind of system in other to draw experimental conclusions about the Seebeck coefficient trend.

**Figure 8:**
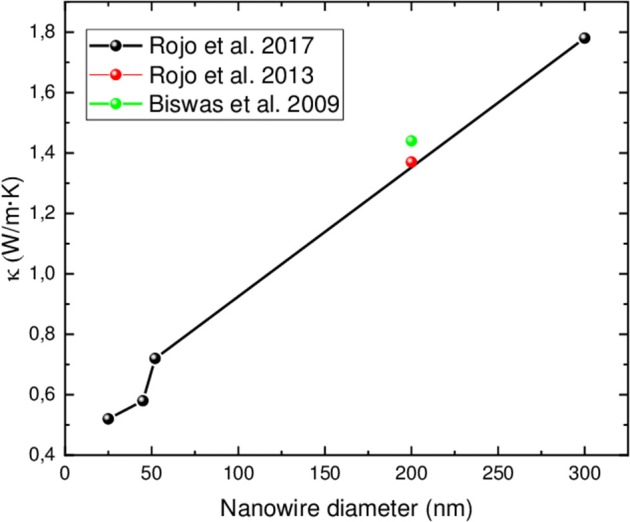
Thermal conductivity as a function of nanowire diameter. Only data for stoichiometric Bi_2_Te_3_ nanowires oriented along [110] direction are compiled in this graph.

Finally, when the thermal conductivity is analyzed taking only into account stoichiometric Bi_2_Te_3_ highly oriented nanowires along [110] direction, a reduction in this magnitude is found when the diameter of the nanowire is decreased. For instance, it has been observed a reduction in thermal conductivity of nanowires when their diameter gets smaller, from 1.6 to 0.8 W/m·K for 120 to 40 nm nanowires diameter, respectively. Most probably, this is due to phonon scattering at the surface of the nanowire, given that the smaller the diameter, the higher the surface to volume ratio (Zhou et al., 2005). Moreover, when the crystallite size decreases in stoichiometric Bi_2_Te_3_ nanowires oriented along [110], the thermal conductivity is reduced from 2.3 to 1.9 W/m·K (Shin et al., 2014). More recently, Martin-Gonzalez's group reported that the thermal conductivity decreased from 1.78 to 0.52 W/m·K for 300 to 25 nm nanowires diameter, respectively (Muñoz Rojo et al., 2017). In that particular work, the Kinetic-Collective model was used to understand such reduction. The main conclusion of this study is that, upon nanowire diameter reduction, the alteration of the mean free path of the low-frequency acoustic phonons is higher. When comparing this model with actual measurements, the agreement was quite remarkable, and thus the reduction in the thermal conductivity of the nanowires can be explained in terms of an increment of phonon scattering in the framework of the Kinetic-Collective model. Finally, Figure 8 shows the thermal conductivity as a function of nanowire diameter for stoichiometric Bi_2_Te_3_ nanowires oriented along [110] direction measured in different works.

Other studies with non-stoichiometric nanowires may lead to different conclusions with respect to the thermal conductivity, if not read with enough detail. For instance, Hill's group observed the opposite tendency, with an increase in the thermal conductivity from 0.1 to 2.4 W/m·K for 40 to 10 nm nanowires diameter, respectively (Ryan et al., 2017). As it is explained in this manuscript, this variation is not due to the size reduction, but to the different stoichiometry of the nanowires (from Bi_11_Te_3_ to Bi_4_Te_5_), and thus this is not comparable with any other study mentioned above.

In summary, by comparing the different works where stoichiometric nanowires oriented in the same direction [110] have been measured, it can be concluded that the electrical conductivity seems to increase as the nanowire diameter decreases. The Seebeck coefficient does not seem to change much when the nanowire diameter decreases, at least with the results reported in the literature and taking into account that the smallest diameter that has been measured so far has been 40 nm. In consequence, it can be concluded that the power factor should increase as the nanowire diameter decreases, at least in the range measured. Finally, it has been confirmed that the thermal conductivity decreases as the nanowire diameter decreases, as it should be expected from the increase in the phonon scattering. Therefore, in a whole, it could be concluded that there is an increase in the figure of merit upon the reduction on the nanowire's diameter.

### Ternary Compounds

Bi_2_Te_3_ can behave as an *n-type* or *p-type* semiconductor depending on its stoichiometry, being a *p-type* semiconductor (positive Seebeck coefficient) for bismuth-rich compositions and an *n-type* semiconductor (negative Seebeck coefficient) for tellurium-rich stoichiometries. Furthermore, it is well known that Bi_2_Te_3_ can turn its *n-type* or *p-type* semiconductor response if it is doped with Se and Sb, respectively. The thermoelectric properties are highly improved in these cases. The optimal composition for (Bi-Te-Se) is close to Bi_2_Te_2.5_Se_0.5_. The ternary compounds of bismuth antimony telluride (Bi-Sb-Te) exhibit a high positive Seebeck coefficient and low thermal conductivity, especially for the compositions close to Bi_0.5_Sb_1.5_Te_3.0_.

### Electrodeposition of Se-Doped Bi-Te Nanowires

In 2003, Stacy's group (Martín-González et al., 2003b) published the first study referent to the electrodeposition of bismuth telluride selenide nanowires. These nanowires were grown at constant potential using AAO templates with two different pore diameters, 50 nm and in commercial Whatman templates (around 200 nm). The composition of the wires was Bi_2.1_Te_2.4_Se_0.5_ with a filling ratio of 75% and they were polycrystalline. In that work, the overall electrochemical reaction was described.

The next study in this material appeared in 2009 by Sima's group (Sima et al., 2009) reporting Bi_2_Te_2.7_Se_0.3_ nanowires obtained at a constant potential in a commercial Whatman template. In 2010, nanowires with a composition of Bi_2_Te_2.7_Se_0.3_ were obtained inside commercial AAO templates (Whatman) and ulterior thermal annealing in Ar atmosphere at 300°C was tested (Li et al., 2010). In 2011, Bi_2_Te_2_Se/Te multilayer nanowires arrays were published. In this case, 60–85 nm diameter and 20 μm length nanowires were obtained by pulsing between BiTeSe and Te reduction potentials (Li X. L et al., 2011). TEM and EDS were performed in the multilayer nanowires in order to confirm both compounds, Bi_2_Te_2_Se and Te.

**Figure 9:**
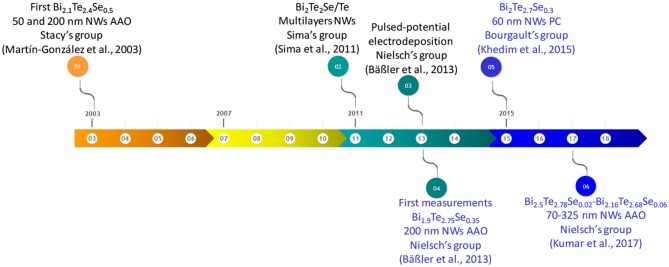
The temporal line of the most relevant achievements of bismuth telluride selenide nanowires grown since 2003. In blue are shown the studies where thermoelectric measurements were performed.

One year later, Bi_2_(Te_0.95_Se_0.05_)_3_ nanowires on 100 nm W/20 nm Ti/Si substrate using AAO templates with 75 nm diameter were electrodeposited (Limmer et al., 2012). Figure 9 shows a temporal line of the most relevant achievements of bismuth telluride selenide nanowires grown since 2003.

In 2013, Nielsch's group (Bäßler et al., 2013) reported pulsed electrodeposition and thermal annealing in Te atmosphere in thermoelectric nanowires of single bismuth telluride selenide nanowires. The pulsed deposition was performed between two potentials using similar pulses than in a previous work for bismuth telluride nanowires (Jongmin et al., 2008). The composition and diameter of the nanowires were Bi_1.9_Te_2.75_Se_0.35_ and 200 nm, respectively. As selenium concentration increases, the power factor increases. The Seebeck coefficient and power factor for as prepared nanowires were found to be −115 μV/K (n-type semiconductor) and 2,660 μW/K^2^·m, respectively. After annealing in Te atmosphere, the Seebeck coefficient and power factor were found to be −115 μV/K and 2,820 μW/K^2^·m, respectively, for the composition aforementioned. After thermal annealing, the Seebeck coefficient does not change, while the power increases due to the increase of electrical conductivity. In 2015, Bourgault's group published a study about the electrodeposition of polycrystalline Bi_2_Te_2.7_Se_0.3_ nanowires with 60 nm in diameter (Khedim et al., 2015). In this work, a strong dependence on chemical composition and morphology with the reduction potential was observed. The Seebeck coefficient measured was −75 μV/K.

In 2017, Nielsch's group (Kumar et al., 2017) reported a study of Bi (Te, Se)_3_ nanowires (ranging from 70 to 325 nm) using similar pulsed conditions as previously (Bäßler et al., 2013). The electrical conductivity is higher for 70 nm than for 325 nm, the smallest Seebeck coefficient was observed for 85-100 nm and the maximum power factor was obtained for 70 nm nanowires. The thermoelectric measurements were performed in a single nanowire. The thermoelectric measurements of Se-doped BiTe nanowires from the literature are collected in Table 3.

**Table 3:** Thermoelectric measurements of Se-doped BiTe nanowires found in the literature.

**Composition and orientation**	**Nanowire diameter (nm)**	**Templates**	**σ (S/cm)**	**S (μV/K)**	**PF (μW/K^2^·m)**	**References**
Bi_2_Te_2.7_Se_0.3_ [1 0 10] and [110]	60	AAO	–	−75	–	Khedim et al., 2015
Bi-rich Bi_2.5_Te_2.78_Se_0.02_- Bi_2.16_Te_2.68_Se_0.06_ Preferential [110]	70–325	AAO	2.7·10^3^–1.9·10^3^	−60 – (−60)	1·10^3^–0.7·10^3^	Kumar et al., 2017
TeSe-rich Bi_1.9_Te_2.75_Se_0.35_ Orientation is not mentioned	200	AAO	2.2·10^3^ ± 0.4·10^3^	−115 ± 2	2.8·10^3^	Bäßler et al., 2013

From the thermoelectric properties of bismuth telluride selenide nanowires (see Table 2), the conclusion that can be extracted is that as nanowire diameter decreases, electrical conductivity slightly increases, similarly as in the case of Bi_2_Te_3_ nanowires (Kumar et al., 2017). No thermal conductivity measurement of these nanowires could be found in literature, so far.

### Electrodeposition Sb-Doped Bi-Te Nanowires

**Figure 10:**
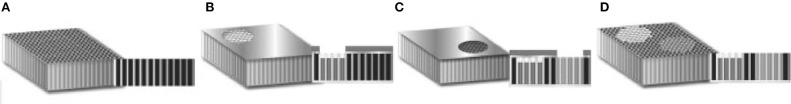
**(A)** alumina templates, **(B)** Bi_2_Te_3_ nanowires electrochemically deposited, **(C)** BiSbTe nanowires electrodeposited and **(D)** Finished thermoelectric device. Reproduced with permission from Lim et al. (2005). Copyright 2005 Advanced Materials.

**Figure 11:**
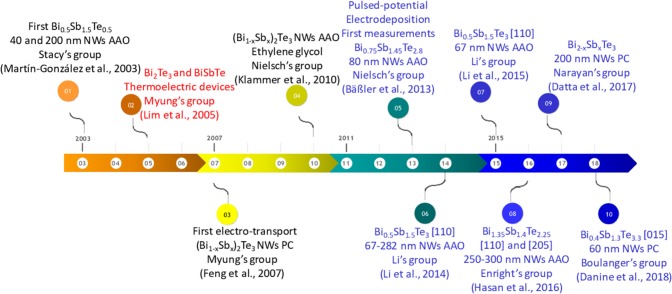
The temporal line of the most important achievements of bismuth antimony telluride nanowires. In blue the studies where thermoelectric measurements were performed is shown. In red is shown the first thermoelectric device based on nanowires.

A historical review on the most relevant results obtained in bismuth antimony telluride nanowires is summarized in the temporal line shown in Figure 11. The temporal line starts in 2003 when Stacy's group (Martín-González et al., 2003a) published for the first time the electrodeposition of polycrystalline Bi_0.5_Sb_1.5_Te_3_ nanowires inside 40 and 200 nm pore diameter AAO templates at a constant potential, describing the general electrochemical reaction. After thermal annealing, the nanowires were textured along [110] direction. Two years later, in 2005, Myung's group (Lim et al., 2005) reported the growth at a constant current density of n-type (Bi_2_Te_3_) and p-type (BiSbTe) nanowires in the same template (43 nm in diameter) in order to develop a thermoelectric device. The composition and crystallographic orientation of the nanowires were not mentioned in the manuscript. Figure 10 shows the thermoelectric nanowire-based fabrication process. Figures 10A–D, show alumina templates, b) Bi_2_Te_3_ nanowires electrochemically deposited, c) Sb-doped BiTe nanowires electrodeposited and d) Finished thermoelectric device.

In 2007, the same group (Feng et al., 2007) published the electron transport properties of (Bi_1−x_Sb_x_)_2_Te_3_ nanowires using polycarbonate membranes of 30–60 nm in pore diameter. When the nanowires were Bi-rich they were preferentially oriented along [110] direction, while in the Sb-rich nanowires the orientation observed was [015]. In 2009, White's group (Li et al., 2009) reported the growth of Bi_0.5_Sb_1.5_Te_3_ nanowires oriented along [110] direction growth at a constant voltage in 80 nm pore diameter PC membranes.

In 2010, Nielsch's group reported the electrodeposition of BiSbTe nanowires of 50 nm in diameter with different compositions (the crystallographic orientation of the nanowires was not mentioned in the manuscript) inside AAO templates, using a non-aqueous solution of ethylene glycol. In 2011, Sima's group (Sima et al., 2011) described (Bi,Sb)_2_Te_3_ nanotubes grown at current pulse plating inside PC membranes performing electrochemical impedance spectroscopy. Two years later, in 2013, Nielsch's group (Bäßler et al., 2013) reported pulsed electrodeposition to fabricate thermoelectric nanowires of Bi_0.75_Sb_1.45_Te_2.8_ with 30 nm diameter. The Seebeck coefficient and power factor were found to be 156 μV/K (p-type semiconductor), the highest reported value for these nanowires to date, and 1750 μW/K^2^·m, respectively. In 2014, Li's group (Li et al., 2014) reported the reduction of thermal conductivity as diameter decreases of Bi_0.5_Sb_1.5_Te_3_ nanowires oriented along [110] direction. The thermal conductivity (0.7-0.89 W/m·K) was measured in a single wire using the 3ω method; these values were lower than that of the bulk material (1.4 W/m·K). The same year, Yen's group (Kuo et al., 2014) reported a work based on pulsed electrodeposition of Bi_0.2_Sb_1.6_Te_3.2_ grown inside AAO templates. Different ratios between *t*_*on*_ and *t*_*off*_ were studied, as well as different diameters and length.

Choa's group (Ng et al., 2014) published a study based on electrodeposition of polycrystalline Bi_x_Sb_y_Te_3_ nanowires obtained with different compositions changing the reduction potential. In 2015, Li's group (Li et al., 2015) reported an improvement of Seebeck coefficient (150 μV/K), reaching similar values than the best previously reported, and electrical conductivity (480 S/cm) when Bi_0.5_Sb_1.5_Te_3_ nanowires with 67 nm in diameter and oriented along [110] direction were grown via pulsed electrodeposition. Moreover, the thermal conductivity was found to be 0.2–0.3 W/m·K, lower than the only other work that had measured it for similar nanowire diameters. The Seebeck coefficient was measured in the nanowires embedded inside the AAO, the electrical conductivity using a four-probe system and the thermal conductivity by the 3ω method.

In 2016, Enright's group (Hasan et al., 2016) published the enhancement of the electrical conductivity (2.5 times higher than bismuth telluride) of Bi_1.35_Sb_1.4_Te_2.25_ nanowires grown via pulsed electrodeposition. The nanowires were highly textured along [110] and [205] directions using AAO templates with 250–300 nm pore diameter. In 2017, Narayan's group (Datta et al., 2017) reported a very low Seebeck coefficient (6.5 μV/K) of polycrystalline Bi_2−x_Sb_x_Te_3_ nanowires grown at constant potential in polycarbonate membranes of 200 nm. In 2018, Boulanger's group (Danine et al., 2018) published 60 nm diameter Bi_0.4_Sb_1.3_Te_3.3_ nanowires grown at constant potential inside polycarbonate membranes oriented along [015] direction. In those nanowires, which presented 60° twins, electrical resistivity, and Seebeck coefficient were measured with values of 128 μΩ·m and 138 μV/K, respectively. The thermoelectric measurements found in the literature of BiSbTe nanowires are collected in Table 4.

**Table 4:** Thermoelectric measurements of Sb-doped BiTe nanowires found in the literature.

**Composition and orientation**	**Nanowire diameter (nm)**	**Template**	**σ (S/cm)**	**S (μV/K)**	**PF (μW/K^2^·m)**	**k (W/m·K)**	**References**
Bi_0.4_Sb_1.3_Te_3.3_ [015] and 60° twins	60	PC	7.5·10^1^	138	142.8	–	Danine et al., 2018
Bi_0.5_Sb_1.5_Te_3_ [110]	67	AAO	4.8·10^2^	150	1,080	0.2–0.3	Li et al., 2015
Bi_0.75_Sb_1.45_Te_2.8_ It is not mentioned	80	AAO	(7.2 ± 6.4) ·10^2^	156 ± 3	(1.7 ± 0.07) ·10^3^	–	Bäßler et al., 2013
Bi_0.5_Sb_1.5_Te_3_ [110]	67–282	AAO	–	–	–	0.7-0.84	Li et al., 2014
Bi_2−x_Sb_x_Te_3_ Polycrystalline	200	PC	–	6.5	–	–	Datta et al., 2017
Bi_1.35_Sb_1.4_Te_2.25_ [110] and [205]	250-300	AAO	2.4·10^3^	–	–	–	Hasan et al., 2016

From the thermoelectric properties of bismuth antimony telluride nanowires (see Table 3), the conclusions that can be extracted are that the Seebeck coefficient does not change much with nanowire diameter. The thermal conductivity slightly decreased from 0.84 to 0.7 W/m·K for 282 to 67 nm nanowires diameter, respectively (Li et al., 2014). These measurements were performed in Bi_0.5_Sb_1.5_Te_3_ nanowires oriented along [110] direction using AAO templates for the electrodeposition.

### Other V-VI Thermoelectrics

Another V-VI compound studied is Sb_2_Te_3_. This material also presents anisotropic behavior in its properties. The figure of merit of bulk antimony telluride single-crystalline in the direction parallel to the *c-axis* of the structure was determined by Antonova et al. (Antonova and Looman, 2005) and it was found to be *zT*_//*c*_ = 0.48, with Seebeck coefficient, electrical conductivity, and thermal conductivity values of 92 μV/K, 0.31 (μΩ·m)^−1^, and 1.63 W/m·K, respectively (Antonova and Looman, 2005). Contrariwise, when the crystal structure was oriented perpendicular to the *c-axis*, the values of the Seebeck coefficient, electrical conductivity, and thermal conductivity were 63 μV/K, 0.82 (μΩ·m)^−1^, and 7.47 W/m·K, respectively, corresponding to a much lower figure of merit of *zT*_⊥*c*_ = 0.13 (Antonova and Looman, 2005).

**Figure 12:**
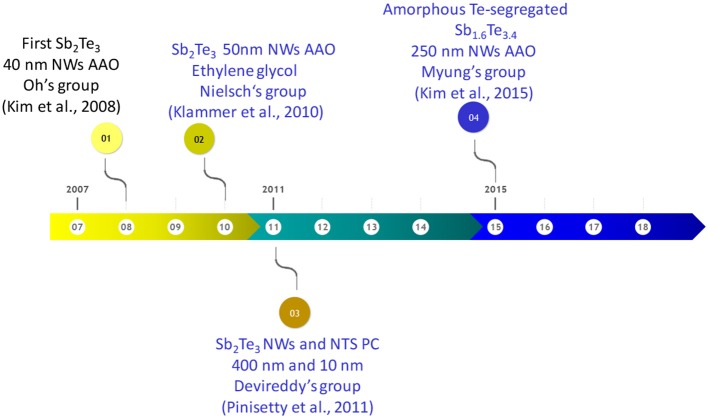
The temporal line of the most relevant achievements of antimony telluride nanowires. In blue are shown the studies where thermoelectric measurements were performed.

The first report on electrodeposition of Sb_2_Te_3_ nanowires can be found in 2008 by Oh's group (Kim et al., 2008). They reported the electrodeposition of polycrystalline Sb_2_Te_3_ nanowires inside commercial AAO template (200 nm pore diameter) using different constant current densities. One year after, the same group published a second paper with similar nanowires, where the power output was measured with a value of 4.8·10^−10^ W (Kim et al., 2009). In 2009, Sb_x_Te_y_ nanowires with different composition were electrodeposited at constant potential using 200 nm pore diameter AAO templates (commercially available Whatmann filters). As reduction potential increases, Te content increases; the length of the wires was ~5–10 μm (Park et al., 2009). Figure 12 shows a temporal line of the most relevant achievements of antimony telluride nanowires.

In 2010, Nielsch's group (Klammer et al., 2010) reported the electrodeposition of antimony telluride nanowires grown in an organic solvent (ethylene glycol) using constant current density and 50 nm pore diameter alumina. The Seebeck coefficient was found to be 21 μV/K. In 2011, Devireddy's group (Pinisetty et al., 2011) published the electrodeposition of nanowires and nanotubes grown inside polycarbonate membranes. Nanowires with 400 nm in diameter and nanotubes with 100 nm in diameter were obtained using different current densities, 10 and 5.5 mA/cm^2^, respectively. The Seebeck coefficient was found to be 359 and 332 μV/K for nanowires and nanotubes, respectively. These values were measured using Harman's technique in nanowires embedded in the membrane. The last study of antimony telluride nanowires grown by electrodeposition can be found in 2015 by Myung's group. In this work, the electrodeposition of amorphous Te-rich antimony telluride (Sb_1.6_Te_3.4_) nanowires with 250 nm diameter using different reduction potentials was carried out. After thermal annealing at 100–200°C, the nanowires were polycrystalline with an increase in the crystallite size from 23 to 28 nm (Kim et al., 2015). The carrier concentration and Seebeck coefficient were found to be 1.1·10^−9^ cm^−3^ and 318 μV/K, respectively. The thermoelectric measurements of SbTe nanowires are collected in Table 5.

**Table 5:** Thermoelectric measurements of SbTe nanowires found in the literature.

**Composition and orientation**	**Nanowire diameter (nm)**	**Template**	**σ (S/cm)**	**S (μV/K)**	**PF (μW/K^2^·m)**	**zT**	**References**
Sb_2_Te_3_ It is not mentioned	50	AAO	–	21	–	–	Klammer et al., 2010
Sb_1.6_Te_3.4_ Polycrystalline	250	AAO	–	318	–	–	Kim et al., 2015
Sb_2_Te_3_ NWs and NTs Polycrystalline	400–100	PC	–	359-332	–	0.09–0.2	Pinisetty et al., 2011

From the available thermoelectric properties of antimony telluride found in the literature (see Table 5), the only conclusion that can be extracted is that the Seebeck coefficient is slightly higher for nanowires than for nanotubes and that an increase in the figure of merit was observed between nanowires (0.09) and nanotubes (0.2) (Pinisetty et al., 2011).

## Interconnected 3D Nanowire Network

**Figure 13:**
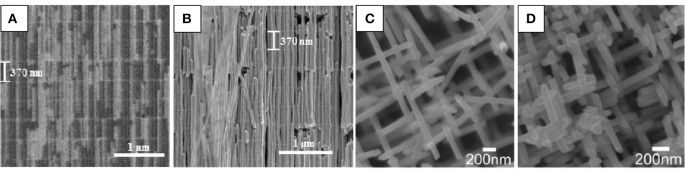
**(A)** Cross-sectional SEM micrograph images of 3D-Bi_2_Te_3_ NW with backscattered electrons; **(B)** SEM micrograph of free-standing 3D Bi_2_Te_3_ nanowire network after AAO template removal. Exhibiting the high degree of interconnectivity and the capacity of being free-standing without the alumina template. Reproduced with permission from Ruiz-Clavijo et al. (2018). Copyright 2018 Nanomaterials. **(C,D)** SEM images of a free-standing 3D network of Sb NWNWs with wire diameters ~100 nm, exhibiting their well–controlled degrees of interconnectivity. Reproduced with permission from Wagner et al. (2016).

One of the latest landmarks in the fabrication of Bi_2_Te_3_ nanowires by electrodeposition is devoted to the fabrication of 3D-interconnected nanowire networks. The first study which reports the electrodeposition of a Bi_2_Te_3_ 3D-interconnected nanowire network was published in 2014 by Martin-Gonzalez‘s group. In this work, a 3D interconnected network of Bi_2_Te_3_ nanowires was obtained by pulsed electrodeposition inside 3D AAO templates (Martín et al., 2014). The nanowire diameters are around 52 nm. In 2018, the same group studied the influence of pulsing (between an applied potential during *t*_*on*_ and current equal to 0 during *t*_*off*_) in the morphology, crystallographic orientation, and composition of the network electrodeposited at 0°C. The crystallographic orientation was controlled changing the reduction potential, and stoichiometric bismuth telluride 3D-nanowires oriented along [110] direction (Ruiz-Clavijo et al., 2018) were fabricated. In order to obtain both the stoichiometric composition and the [110] crystallographic orientation, pulsed current-voltage electrodeposition was applied between a density current of 0 mA/cm^2^ for 0.1 s and difference potential for 1s. Figure 13 shows cross-sectional SEM images of 3D-Bi_2_Te_3_ NW embedded into the AAO template (Figure 13A) and the free-standing structure, after selectively removing the AAO template (Figure 13B). Additionally, the thermal conductivity of stoichiometric bismuth telluride 3D-nanowires oriented along [110] direction and 52 nm in diameter was measured by Martin-Gonzalez‘s group (Abad et al., 2016b). A value of 0.58 ± 0.22 W·m^−1^K^−1^ was found, which shows a reduction in the thermal conductivity value, with respect to 1D nanowires of the same diameter (0.72 ± 0.37 W·m^−1^K^−1^) (Muñoz Rojo et al., 2017). One may think that the reduction in the thermal conductivity between the 3D vs. the 1D could be due to a possible effect of phononic crystal, because of the internal structure of the 3D network. But, before reaching that conclusion, further experiments need to be done.

In 2016, Toimil-Molares's group reported a Sb 3D network inside 3D-PC membranes with 100 nm in diameter and 30 μm in length using pulsed-potential deposition. Although this material is not a V-VI compound, we consider the work of high importance to be mentioned here. These nanowires presented two crystallographic directions, [110] and [104] (Wagner et al., 2016). Furthermore, in this study, thermoelectric measurements using a cross-plane steady-state method was performed in a 3D network with 30 μm in length. The effect of the interconnectivity of the nanowires network is shown. Figures 13C,D show SEM images of 3D-Sb NW network free-standing.

The principal differences between both networks are that the vertical spacing of 3D-AAO nanostructures can be controlled very accurately since it is proportional to the time of the pulses applied during their fabrication, so they can be tailored as desired. The wires are perpendicular to the template surface and they have horizontal interconnections at 90 degrees to the vertical channels (see Figures 13A,B). These interconnections are perfectly aligned and form a plane of interconnections. This 3D-AAO can also be obtained in non-planar surfaces like in cylinder or wires (Resende et al., 2016). In addition, these 3D-AAO nanostructures could be considered phononic crystals, due to the periodic structures that can be produced inside the membranes. These phononic crystal nanostructures would allow the reduction of the thermal conductivity thanks to a new approach for changing the thermal transport based on the concept of nanophononic metamaterials (NPMs) by introducing nanoscale local resonators. In this case, the interconnections distances could be adjusted to enable unique subwavelength properties and killing the propagation of certain phonon wavelengths.

In the case of 3D-PC nanostructures, this spacing cannot be controlled since the ion irradiation is a random process. Only the average density of nanowires, the relative angle with the surface and the pore diameter can be controlled by the influence of the ions during the template fabrication and the etching time during the opening of the pores. In this case, the 3D-PC the nanowires are oriented 45 degrees from the template surface and not complete planes of interconnected nanowires were obtained as can be seen in Figures 13C,D.

These 3D-AAO and the 3D-PC membranes open a new avenue to obtain macroscopic hierarchical 3D nanowire networks that will have all the benefits of the nanowires in a macroscopic system that can be handled with tweezers and without any of the drawbacks of having the template in the structure.

## Conclusions

In this review, it has been shown that stoichiometric Bi_2_Te_3_ nanowires oriented along [110] direction have been grown by electrodeposition with different diameters (25–400 nm) up to date. The smallest nanowire diameter of these nanowires was found to be ~15 nm. Moreover, all the thermoelectric properties (electrical conductivity, Seebeck coefficient, and thermal conductivity) have been measured for different diameters. The electrical conductivity seems to increase in the different works as nanowire diameter decreases for stoichiometric Bi_2_Te_3_ nanowires oriented along [110] direction. The change in the Seebeck coefficient can be explained by other factors than by nanowire diameter reductions. And the thermal conductivity decreases as nanowire diameter decreases. Additionally, in the case of Se-doped BiTe, higher electrical conductivity and Seebeck coefficient than Bi_2_Te_3_ have been reported. And, in the case of Sb-doped BiTe, higher Seebeck coefficient and lower thermal conductivity have been published.

In summary, the most important conclusion is that the nanowires should be grown with small diameter (20–25 nm), given that the electrical conductivity seems to increase for smaller diameters and the thermal conductivity decreases as the diameter decreases. It is worth to note that the different studies found in the literature were done using different tellurium sources and that this can explain the variations on the Seebeck coefficients measured more than the effect on the size reduction.

Regarding the measurements, it is important to highlight that there is a lack of metrology at the nanoscale, even though many different phenomena (electrical contacts, accurate temperature gradient measurement at the nanoscale, stoichiometry of the nanowires, crystalline orientation, tellurium source used, the number of twins, etc.) play an important role in the dispersion of the measurements. Each article uses their own system to measure at the nanoscale and no round-robin experiments between the different setups have been made to date.

Recently, the development of 3D network nanowires has opened a new field to investigate the thermoelectric properties of these nanostructures. These 3D network nanowires are the future to develop more efficient thermoelectric materials. Nevertheless, different questions are still open in the field. One open question that it is necessary to gain understanding on is the reason why the electric conductivity increases as the diameter decreases, when the theory predicts that this behavior only should be observed in quantum confinement regimes (for nanowire diameters lower than 5–10 nm). Another open question is what will be the value of the Seebeck coefficient for nanowires with a diameter smaller than 40 nm, because in theoretical studies performed to date some phenomena, which will be relevant at such diameters, were not included, such as Dirac superficial phenomenon. Or, the fabrication of a phononic crystal, to cite some.

Therefore, the research in thermoelectric nanowires is nowadays a hot topic in many ways. They provide a more than reasonable way of improving the current performance of thermoelectric materials, but they need a great research effort in three main directions to exploit all their potential: in their fabrication techniques, to go to lower diameters with stoichiometric and properly oriented materials; in the characterization techniques, to asses an accurate way of measuring transport properties in nanostructures and better metrology at the nanoscale; and in the theoretical understanding of the phenomena taking place in these nanostructures.

## Author Contributions

All authors listed have made a substantial, direct and intellectual contribution to the work, and approved it for publication.

### Conflict of Interest Statement

The authors declare that the research was conducted in the absence of any commercial or financial relationships that could be construed as a potential conflict of interest.
